# Relationship between risk of oral frailty and awareness of oral frailty among community-dwelling adults: a cross-sectional study

**DOI:** 10.1038/s41598-023-50818-6

**Published:** 2024-01-03

**Authors:** Koichiro Irie, Yuki Mochida, Nandin Uchral Altanbagana, Shinya Fuchida, Tatsuo Yamamoto

**Affiliations:** 1 Department of Preventive Dentistry and Dental Public Health, Kanagawa Dental University, 82 Inaoka-cho, Yokosuka, Kanagawa 238-8580 Japan; 2 Department of Education Planning, Kanagawa Dental University, 82 Inaoka-cho, Yokosuka, Kanagawa 238-8580 Japan

**Keywords:** Diseases, Health care, Risk factors, Signs and symptoms

## Abstract

We aimed to investigate the relationship between the risk of oral frailty and awareness of oral frailty among Japanese adults in an adult dental health field study conducted in Kanagawa Prefecture. Questionnaire data from a total of 5051 individuals (1907 males, 3144 females; mean age; 59.9 years) were used. The risk of oral frailty was assessed using the Oral Frailty Index-8. Of the participants, 1418 (28.1%) had a high risk of oral frailty and 1495 (29.6%) had knowledge of oral frailty. Logistic regression analysis indicated that the risk of oral frailty was significantly associated with awareness of oral frailty. We further found that awareness of oral frailty was significantly related to gender (female), age (20–39 compared to 70–79, ≥ 80), residential areas (Yokohama compared to Kawasaki, Sagamihara), exercise habits (yes), eating a balanced diet (yes), consciousness of oral health (yes), risk of oral frailty (low) and outpatient category (hospital visit). For groups with low levels of awareness obtained from the results of this study, it is necessary to consider the means of accessibility and increase awareness further.

## Introduction

Eating is the foundation of human life. The ability of older people to eat is supported by a wide variety of factors related to tooth and oral functions, such as the number of teeth present, masticatory strength, swallowing function, and occlusal support^1^. To increase awareness of the importance of oral function in the Japanese population, the concept of oral frailty has been introduced. Oral frailty, as defined by the Japan Dental Association, is a series of phenomena and processes characterized by vulnerability of oral health status due to age-related changes in different oral health conditions (number of teeth, oral hygiene, and oral functions)^2^.

Oral frailty provides a warning to avoid the following negative repercussions: neglecting slight declines in oral function and ignoring such conditions without taking appropriate measures eventually leads to deterioration of oral function, impairment of eating function, and even deterioration of physical and mental functions^3^. Oral frailty is a risk factor for frailty and mortality^3^. To prevent oral frailty, active oral care, regular dental check-ups and a healthy lifestyle have been shown to mitigate the effects of oral frailty and contribute to improving the quality of life of older people^4^.

Coordinating the prevention of oral frailty, by detecting early symptoms (trivial signs) before significant declines in oral function, and recognizing the importance of the basic rules of thumb of “walk well, chew well, and eat well”^5,6^. Oral health knowledge is considered to be crucial for developing healthy behavior, and it has been shown that there is an association between increased knowledge and better oral health^7,8^. Optimum health-related practices are more likely to be adopted if an individual feels a better sense of control over their health and has a better understanding of diseases and their etiology. One method for prevention is to improve community awareness regarding the promotion of healthy behaviors and the influence of self-effective methods in preventing disease. For example, awareness of the 8020 Movement, a Japanese social movement to keep at least 20 of one’s own teeth to the age of 80 years, was significantly associated with improved regular dental visits^9^. However, it is unclear whether awareness of oral frailty has any affect.

Recently, to help screen older people at risk of oral frailty in community settings, an eight-item questionnaire called, the Oral Frailty Index-8 (OFI-8) was proposed^10^. Assessing the individual risk of oral frailty in patients with limited access to dental can help increase oral health literacy and awareness of oral frailty in the community. Therefore, we hypothesized, herein, that awareness of oral frailty may affect the risk of oral frailty. Thus, this cross-sectional study aimed to investigate the relationship between the risk of oral frailty using the OFI-8 questionnaire and awareness of oral frailty among Japanese adults in an adult dental health field study conducted in Kanagawa Prefecture. Our secondary aim was to investigate the factors influencing awareness of oral frailty to inform future oral health policies.

## Methods

### Study population

This cross-sectional study employed data from the adult dental health field study conducted in Kanagawa Prefecture. The corresponding author signed a memorandum of understanding with Kanagawa Prefecture regarding use of the screening data. The participants were first-time patients residing in the prefecture who visited a dental clinic or had a home -visit by a member of the Kanagawa Dental Association. They participated in the study between June 2020 and March 2021 to evaluate the actual status of oral health in Kanagawa Prefecture.

The study included individuals ≥ 20 years of age. Patients with missing data surrounding items representing the risk of oral frailty and other variables were excluded. Of the 5918 individuals who underwent assessment, 867 were excluded based on the exclusion criteria. A total 5051 people (1907 males, 3144 females; mean age; 59.9 years old; standard deviation: 18.7 years) were analyzed in the examination.

### Definition and assessment of OFI-8

The presence or absence of risk of oral frailty was evaluated based on eight items, which was a modification of the method described by Tanaka et al^10^. The OFI-8 is an eight-item screening questionnaire that integrates oral health-related behaviors and frailty concepts. OFI-8 doubles the score of the three higher priority items for the most important elements of oral frailty (“tooth loss,” “subjective chewing difficulties,” and “subjective swallowing difficulties”). Total OFI-8 scores ranged from 0 to 11 points, with higher scores indicating poorer oral health. Each question was extracted from a questionnaire that is widely used in Japan. All items were scored as follows:

1. “Do you have any difficulties eating tough foods compared to 6 months ago?” (Yes, 2 points).
2. “Have you choked on your tea or soup recently?” (Yes, 2 points).
3. “Do you use dentures?” (Yes, 2 points).
4. “Do you often have a dry mouth?” (Yes, 1 point).
5. “Do you go out less frequently than you did last year?” (Yes, 1 point).
6. “Can you eat hard foods like squid jerky or pickled radish?” (No, 1 point).
7. “How many times do you brush your teeth in a day?” (< 3 times/day, 1 point).
8. “Do you visit a dental clinic at least annually?” (No, 1 point).

The higher the OFI-8 score, the higher the risk of oral frailty, that is, 0–2 points indicates low risk; 3 points, moderate risk; and 4–11 points, high risk. A score of 4 ≥ points indicates the necessity of a dental check-up, as older adults with such a score are at high risk of new-onset oral frailty and new long-term care needs certification^10^. Therefore, the OHI-8 score of 4 ≥ points were defined as high risk of oral frailty in the present study^11^.

### Questionnaire survey

Participants completed a self-administered questionnaire. Data regarding participants `age, gender, residential areas (Yokohama, Kawasaki, Sagamihara, Yokosuka/Miura, Shonan-tobu, Shonan-seibu, Kenoh, Kensei), exercise habits (yes or no), smoking habits (yes or no) and outpatient category (home visit or hospital visit) were obtained. Ages were categorized into 20–39, 40–49, 50–59, 60–69, 70–79, and ≥ 80 years. Medical history including diabetes mellitus, heart disease, and cerebrovascular disease was obtained during a medical interview using a standardized questionnaire.

We assessed participant’s awareness of oral frailty by asking whether they knew of oral frailty. Respondents who answered ‘I know what it means’ or ‘I know the term’ were categorized as ‘yes’; respondents participants were also asked “Do you eat a balanced diet?” (Yes or no), and “Are you consciousness of oral health?” (Yes or no).

### Statistical analysis

Descriptive statistics were used to characterize the study population and compare groups at high risk of oral frailty and with awareness of oral frailty. Student’s *t*-test, Mann–Whitney *U* statistic, or the Chi-squared test were used where appropriate.

Odds ratios and 95% confidence intervals were calculated using logistic regression. For the risk of oral frailty, according to previous studies^6,10^ and descriptive statistics (*p* < 0.10), the following variables were selected based on confounder variables: gender, age, diabetes mellitus, heart disease, pneumonia, cerebrovascular disease, exercise habits, eating a balanced diet, consciousness of dental health, awareness of oral frailty and outpatient category. For awareness of oral frailty, from descriptive statistics (*p* < 0.10), the following variables were selected based on confounder variables: gender, age, residential area, diabetes mellitus, cerebrovascular disease, exercise habits, smoking habits, eating a balanced diet, consciousness of oral health, risk of oral frailty, and outpatient category. Statistical analysis was performed using the software package IBM SPSS Statistics (v. 29.0, SPSS Japan Inc.) at a significance level of 0.05.

### Ethics approval

All data used in the analysis were anonymous and the requirement for informed consent was waived based on the Ethics Guidelines for Medical and Biological Research Involving Human Subjects in Japan. The corresponding author signed a memorandum of understanding with Kanagawa Prefecture regarding use of the survey data. Kanagawa Prefecture issue clearance for secondary analysis of survey data (approval No. KEU2642). The study was carried out in accordance with the revised Declaration of Helsinki.

## Results

Of the total number of participants, 3633 (71.9%) had a low risk of oral frailty, and 1418 (28.1%) had a high risk of oral frailty. Comparisons of participant characteristics according to the risk of oral frailty are presented in Table 1. A high risk of oral frailty was significantly associated with older age, diabetes mellitus (yes), heart disease (yes), pneumonia (yes), cerebrovascular disease (yes), exercise habits (no), eating a balanced diet (no), consciousness of oral health (no), awareness of oral frailty (no), and outpatient category (home visit).

**Table 1:** Characteristics of the study participants and subjects with high risk of oral frailty.

Variables	Categories	Total	High risk of oral frailty (n = 1418)		*p* value
		n = 5051	n	%	
Gender	Male	1907	532	27.9	0.846
	Female	3144	886	28.2	
Age, years	20–39	803	49	6.1	< 0.001
	40–49	732	73	10.0	
	50–59	834	141	16.9	
	60–69	860	245	28.5	
	70–79	999	403	40.3	
	≥ 80	823	507	61.6	
Residential areas	Yokohama	1971	570	28.9	0.725
	Kawasaki	1029	283	27.5	
	Sagamihara	322	96	29.8	
	Yokosuka/Miura	501	143	28.5	
	Shonan-tobu	387	108	27.9	
	Shonan-seibu	242	64	26.4	
	Kenoh	436	107	24.5	
	Kensei	163	47	28.8	
Diabetes mellitus	Yes	129	75	58.1	< 0.001
	No	4922	1343	27.3	
Heart disease	Yes	116	78	67.2	< 0.001
	No	4935	1340	27.2	
Cerebrovascular disease	Yes	75	53	70.7	< 0.001
	No	4976	1365	27.4	
Exercise habits	Yes	3573	880	24.6	< 0.001
	No	1478	538	36.4	
Smoking habits	Yes	973	297	30.5	0.062
	No	4078	1121	27.5	
Eating a balanced diet	Yes	2163	567	26.2	0.011
	No	2888	851	29.5	
Consciousness of oral health	Yes	4588	1192	26.0	< 0.001
	No	463	226	48.8	
Awareness of oral frailty	Yes	1495	279	18.7	< 0.001
	No	3556	1139	32.0	
Outpatient category	Home visit	685	380	55.5	< 0.001
	Hospital visit	4366	1038	23.8	

Table 2 presents the results of binomial regression analysis with the risk of oral frailty as the dependent variable. High risk of oral frailty was significantly related to gender (male), age (20–39 compared to 40–49, 50–59, 60–69, 70–79, ≥ 80), diabetes mellitus (yes), heart disease (yes), exercise habits (no), eating a balanced diet (no), consciousness oral health (no), awareness of oral frailty (no) and outpatient category (home visit) even after adjusting for variables.

**Table 2:** Factors associated with the high risk of oral frailty by binomial logistic regression analysis with stepwise variable selection.

Independent variables	ORs (95% CI)	*p* value
Gender (Male) (ref: Female)	1.22 (1.05–1.41)	0.009
Age (years) (ref: 20–39)		
40–49	1.71 (1.17–2.50)	0.006
50–59	3.33 (2.36–4.71)	< 0.001
60–69	6.80 (4.88–9.48)	< 0.001
70–79	11.33 (8.18–15.70)	< 0.001
≥ 80	20.05 (14.29–28.13)	< 0.001
Diabetes mellitus (Yes) (ref: No)	2.02 (1.35–3.04)	< 0.001
Heart disease (Yes) (ref: No)	1.91 (1.21–2.99)	0.005
Cerebrovascular disease (Yes) (ref: No)	1.79 (1.03–3.12)	0.040
Exercise habits (No) (ref: Yes)	1.60 (1.36–1.87)	< 0.001
Eating a balanced diet (No) (ref: Yes)	1.40 (1.20–1.63)	< 0.001
Consciousness of oral health (No) (ref: Yes)	2.08 (1.65–2.62)	< 0.001
Awareness of oral frailty (No) (ref: Yes)	1.54 (1.31–1.82)	< 0.001
Outpatient category (Home visit) (ref: Hospital visit)	1.55 (1.27–1.90)	< 0.001

*CI* Confidence interval, *OR* Odds ratio.

Dependent variable: risk of oral frailty (1: high, 0: low).

Independent variables entered the model were gender, age, diabetes mellitus, heart disease, cerebrovascular disease, exercise habits, eating a balanced diet, consciousness of dental health, awareness of oral frailty, and outpatient category.

Table 3 compares participant characteristics according to their awareness of oral frailty, 1495 (29.6%) had awareness of oral frailty. Awareness of oral frailty (yes) was significantly affected by gender (female), age, residential area, diabetes mellitus (no), cerebrovascular disease (no), exercise habits (yes), smoking habits (no), eating a balanced diet (yes), consciousness of oral health (yes), risk of oral frailty (low), and outpatient category (hospital visits).

**Table 3:** Characteristics of the study participants and subjects with having awareness of oral frailty.

Variables	Categories	Total	Having awareness of oral frailty (n = 1495)		*p* value
		n = 5051	n	%	
Gender	Male	1907	446	23.4	< 0.001
	Female	3144	1049	33.4	
Age, years	20–39	803	274	34.1	< 0.001
	40–49	732	220	30.1	
	50–59	834	289	34.7	
	60–69	860	294	34.2	
	70–79	999	276	27.6	
	≥ 80	823	142	17.3	
Residential areas	Yokohama	1971	607	30.8	< 0.001
	Kawasaki	1029	254	24.7	
	Sagamihara	322	78	24.2	
	Yokosuka/Miura	501	151	30.1	
	Shonan-tobu	387	128	33.1	
	Shonan-seibu	242	83	34.3	
	Kenoh	436	148	33.9	
	Kensei	163	46	28.2	
Diabetes mellitus	Yes	129	22	17.1	0.001
	No	4922	1473	29.9	
Heart disease	Yes	116	31	26.7	0.538
	No	4935	1464	29.7	
Cerebrovascular disease	Yes	75	14	18.7	0.041
	No	4976	1481	29.8	
Exercise habits	Yes	3573	1137	31.8	< 0.001
	No	1478	358	24.2	
Smoking habits	Yes	973	246	25.3	0.001
	No	4078	1249	30.6	
Eating a balanced diet	Yes	2163	770	35.6	< 0.001
	No	2888	725	25.1	
Consciousness of oral health	Yes	4588	1422	31.0	< 0.001
	No	463	73	15.8	
Risk of oral frailty	Low: 0–3	3633	1216	33.5	< 0.001
	High: 4–11	1418	279	19.7	
Outpatient category	Home visit	685	114	16.6	< 0.001
	Hospital visit	4366	1381	31.6	

Table 4 shows results of the binomial regression analysis with awareness of oral frailty as the dependent variable. Awareness of oral frailty was significantly related to gender (female), age (20–39 compared to 70–79, ≥ 80), residential areas (Kawasaki, Sagamihara), exercise habits (yes), eating a balanced diet (yes), consciousness of oral health (yes), risk of oral frailty (low), and outpatient category (hospital visit) even after adjusting for variables.

**Table 4:** Factors associated with awareness of oral frailty by binomial logistic regression analysis with stepwise variable selection.

Independent variables	ORs (95% CI)	*p* value
Gender (Female) (ref: Male)	1.60 (1.40–1.83)	< 0.001
Age (years) (ref: 20–39)		
40–49	0.82 (0.66–1.02)	0.070
50–59	1.03 (0.83–1.27)	0.811
60–69	1.05 (0.85–1.30)	0.652
70–79	0.78 (0.63–0.97)	0.026
≥ 80	0.56 (0.43–0.72)	< 0.001
Residential areas (ref: Yokohama)		
Kawasaki	0.71 (0.60–0.85)	< 0.001
Sagamihara	0.69 (0.52–0.91)	0.009
Yokosuka/Miura	0.96 (0.77–1.20)	0.716
Shonan-tobu	1.08 (0.85–1.37)	0.543
Shonan-seibu	1.14 (0.85–1.53)	0.381
Kenoh	1.10 (0.88–1.39)	0.395
Kensei	0.86 (0.60–1.24)	0.425
Exercise habits (Yes) (ref: No)	1.22 (1.05–1.41)	0.010
Eating a balanced diet (Yes) (ref: No)	1.52 (1.33–1.74)	< 0.001
Consciousness of oral health (Yes) (ref: No)	1.62 (1.24–2.12)	< 0.001
Risk of oral frailty (Low) (ref: High)	1.56 (1.33–1.84)	< 0.001
Outpatient category (Hospital visit) (ref: Home visit)	1.63 (1.29–2.05)	< 0.001

*CI* Confidence interval, *OR* Odds ratio.

Dependent variable: awareness of oral frailty (1: yes, 0: no).

Independent variables entered the model were gender, age, residential area, diabetes mellitus, cerebrovascular disease, exercise habits, smoking habits, eating a balanced diet, consciousness of dental health, risk of oral frailty, and outpatient category.

## Discussion

Herein, we investigated whether the risk of oral frailty is associated with awareness of oral frailty in community dwelling Japanese adults. Our results showed that the risk of oral frailty was significantly associated with awareness of oral frailty. We further found that awareness of oral frailty was to be influenced by factors such as gender, age, residential area, exercise habits, eating a balanced diet, consciousness of oral health, risk of oral frailty and outpatient category. This is the first study to examine the association between risk of oral frailty and awareness of oral frailty. These results suggest the importance of disseminating oral frailty awareness particularly to populations with low awareness levels, thus preventing oral frailty. It is especially important to inform oral frailty high-risk individuals in dental clinics, since the population of this study was dental clinic patients.

Our results showed that 29.6% of participants recognized oral frailty. The target of 50%, set by the Japanese Dental Association by 2025 has not yet been reached. The high risk of oral frailty among those with no awareness of it indicates the importance of raising awareness. For groups with low levels of awareness obtained from the results of this study, it is necessary to consider the means of accessibility and to further increase awareness. Most oral health problems in older adults can be prevented through routine oral healthcare^12^. Surprisingly, individuals at high risk of oral frailty were also found in the younger age group. Therefore, there is a need to raise awareness of the importance of oral function not only among the older people but also among the younger population. Thus, healthcare providers should develop educational programs that provide detailed oral health knowledge that can be directly linked to oral health behavior. It is necessary to devise and implement not only one-time education programs, but also to plan for continuous oral health education.

The risk of oral frailty is also associated with age, diabetes mellitus, heart disease, cerebrovascular disease, exercise habits, and balanced diet. Previous studies have shown that oral health status is associated not only with aging^13,14^, but also with systemic disease^15^. Oral health literacy, including exercise habits and balanced diet, also affects oral health status^16^. Oral health problems such as tooth loss, decline in swallowing function, and low tongue pressure are interrelated and no single oral health condition assessment can capture these phenomena^3^. Oral frailty is a series of processes that lead to age-associated changes in various oral conditions, such as the number of teeth present, oral hygiene, and oral dysfunction, together with a decreased interest in oral health^2^. Therefore, early recognition of declining oral health status and promotion of treatment of declining oral function, especially in high-risk populations, may be effective in preventing oral frailty.

The following reasons can be considered as possible reasons why awareness of oral frailty was associated with risk of oral frailty. Poor oral knowledge has been proposed as a causal factor for disparities in oral health outcomes^17^. Oral health education is effective in modifying oral health behavior^18,19^. For instance, knowledge of dental flossing was positively associated with the use of dental floss and regular dental checkups^20^. In addition, some reports have suggested that acquiring dental knowledge from dental clinics effectively induces good oral health behaviors, which contributes to the achievement and maintenance of superior periodontal status^7,20,21^. Furthermore, awareness of the 8020 movement was significantly associated with regular dental visits^9^. They may be willing to do regular dental visits to retain 20 or more of one`s own teeth. Altogether, there is general agreement that good dental awareness and good oral health behavior are correlated.

In addition, factors related to awareness of oral frailty were gender, age, residential area, exercise habits, eating a balanced diet, consciousness of oral health, risk of oral frailty, and outpatient category. A previous study showed that females possessed greater knowledge of oral health and a more positive attitude toward dental visit than males^9^. Furthermore, older age is associated with lower levels of oral health knowledge^22^. Oral health knowledge also varies regionally owing to a combination of factors, including cultural practices, access to health care, and educational initiatives^23,24^. Regarding residential area, there were differences Kawasaki are Sagamihara compared to Yokohama. Differences in health policies between the regions may have an impact. To increase residents′ awareness of oral frailty, municipalities are conducting various projects such as holding lectures and training resident volunteers. Yokohama is a large city with a large budget and may have an extensive program related to public awareness. Further analysis is needed to obtain this information in the future. Additionally, oral health literacy, including exercise habits and eating a balanced diet is related to oral health knowledge^25^. Therefore, our results are consistent with the findings of a previous study and provide useful information about raising awareness regarding oral frailty.

The present study had certain limitations. First, causal associations could not be determined because this was a cross-sectional study. Prospective follow-up studies are required to confirm these findings. Second, other possible confounders, such as life style^26^, education level^27^, social capital^28^, sense of coherence^29^ and self-efficacy^30^ were not included in this study. Third, it is not known where they obtained information about oral frailty. Clarifying the source of the information is an issue for future study, as it is important for the future dissemination of oral frailty. It is also unclear to what extent those who reported “know” knew the specific of the term. It is important to assess specific health behaviors rather than merely knowing the terminology. Forth, we evaluated the risk of oral frailty using OFI-8 across various age group. The appropriateness of using OFI-8 in young adults has not yet been studied. The reason why we did not limit the subjects in this study to those aged 65 years and older was to convey the importance of taking measures to prevent oral frailty from a young adulthood. The fact that the percentage and ORs of the high risk of oral frailty increase age (Tables 1 and 2) suggests that OFI-8 may be somewhat adaptable to those under 65 years of age. Further studies are required to confirm the appropriateness of using OFI-8 in young adults.

## Conclusions

Risk of oral frailty was significantly associated with awareness of oral frailty. Additionally, awareness of oral frailty was found to be influenced by factors such as gender, age, residential area, exercise habits, eating a balanced diet, consciousness of oral health, risk of oral frailty, and outpatient category even after adjusting for possible confounders.

## Data Availability

The data that support the findings of this study are available from Kanagawa Prefecture but restrictions apply to the availability of these data, which were used under license for the current study, and so are not publicly available. Data are not available from the corresponding author. Those who wish to obtain a license, i.e., those who wish to make secondary use of the data, submit an application for the provision of questionnaire information to the Kanagawa Prefectural Governor. In addition to the applicant’s information, the application form includes the purpose, method, and period of data use, as well as the method of publication of the results.
