# Author Correction: Relationship between risk of oral frailty and awareness of oral frailty among community-dwelling adults: a cross-sectional study

**DOI:** 10.1038/s41598-025-89192-w

**Published:** 2025-02-11

**Authors:** Koichiro Irie, Yuki Mochida, Nandin Uchral Altanbagana, Shinya Fuchida, Tatsuo Yamamoto

**Affiliations:** 1 Department of Preventive Dentistry and Dental Public Health, Kanagawa Dental University, 82 Inaoka-cho, Yokosuka, Kanagawa 238-8580 Japan; 2 Department of Education Planning, Kanagawa Dental University, 82 Inaoka-cho, Yokosuka, Kanagawa 238-8580 Japan

Correction to: *Scientific Reports* 10.1038/s41598-023-50818-6, published online 03 January 2024

The original version of this Article contained an error in Table 1 where total value for “Awareness of oral health” was incorrect,

“3356”

now reads:

“3556”

The original Article has been corrected.

